# Memento for interprofessional learning

**DOI:** 10.1007/s00428-020-02803-x

**Published:** 2020-04-08

**Authors:** Patricia J. T. A. Groenen, A. W. Langerak, F. Fend, J. H. J. M. van Krieken

**Affiliations:** 1 Dept. of Pathology, Radboud University Medical Centre Nijmegen, Geert Grooteplein Zuid 10, P.O Box 9101, 6500 HB Nijmegen, The Netherlands; 2 Department of Immunology, Laboratory for Medical Immunology, Erasmus MC, University Medical Center, 3015 CN Rotterdam, The Netherlands; 3 Institute of Pathology and Neuropathology, University Hospital Tuebingen/Eberhard-Karls-University, 72076 Tuebingen, Germany

**Keywords:** Molecular diagnostics, Pathology, Oncology, Learning, Interprofessional

## Abstract

The vast increase of technical, diagnostic, and treatment possibilities and deepened understanding of molecular biology has revolutionized diagnosis and treatment of cancer and thus has great impact on pathology. Different professionals are responsible for proper evaluation of the results and their translating into an accurate diagnosis and appropriate treatment. Next to expertise, a close interaction between clinical molecular biologists, pathologists, and oncologists is required; it is crucial that these professionals speak “the same language.” Key to this is communication skills and creating possibilities for collaboration in a meaningful context. Here, we present an interprofessional, educational workshop model and we describe the parameters that contribute to effective learning by specialists.

A modern pathology laboratory has practicing pathologists and clinical molecular biologists each with their own expertise, role, and responsibility. Exchange of relevant information across both professionals is essential to properly apply and integrate test results for state-of-the-art pathology reporting. Here, we stress the importance of interprofessional learning.

Implementation of complex tests and understanding the impact and potential is a difficult process. Clonality analysis of antigen receptor gene rearrangements in lymphoma is such a complex test, which has gained wide acceptance through well-cited publications, but causes problems in routine practice. It was decided to set up an educational workshop to promote the correct use of clonality analysis so that patients are not over- or under-treated. Because it turned out that the integration of knowledge from different disciplines (histopathology and molecular biology) was the core problem, a cased-based, interprofessional workshop was chosen.

The annual workshop (http://www.euroclonality.org/workshop/) is a real hands-on workshop; most of the time is taken for case discussions. Participants are only accepted when they come as a team representing different disciplines from one center: a pathologist and a molecular biologist. Each team has to bring at least one illustrative case that created problems and present the pathological findings, clinical context, and the molecular data. This guarantees a minimum level of experience of the participants and sufficient knowledge to be actively involved in the discussions. There are also educational lectures given by the faculty, that is from various disciplines as well.

To evaluate the learning process, a questionnaire form is filled out by the participants. This revealed important parameters for effective learning:

1. A safe environment: the workshop takes place in a small group. The small group, the easily accessible faculty, and the shared meals (lunches and dinners) provide a safe learning environment, which is an important parameter for effective adult learning [1, 2].
2. Interprofessional approach: the pathologist and the molecular biologist learn together. There is exchange of relevant information, from both sides. The professionals feel their perspective valued. This approach entails translation between the different “professions”, thereby learning “to speak the same language” [3].
3. A meaningful context: the workshop is focused on discussion of own cases, which is a powerful enhancer of learning [4]. Similar cases might have been seen by other participants in their practice and are therefore educational for the entire group.
4. Immediate feedback is given by the faculty at multiple levels: (a) about the results and interpretation, (b) addressing deeper learning covering new applications or understanding of the pathobiology, (c) addressing the way the participants will regulate their continuous learning process. Providing feedback contributes greatly to the learning process [5].

In oncology diagnostics, there are many fascinating novel developments. Medical professionals should maintain lifelong learning skills and drive their own educational process to develop new competencies. Our workshop has been organized for more than 10 years, is very well appreciated, and has been effective to build new competences. Our experience may assist other organizers in developing similar training programs.
